# What Frequency of Technical Activity Is Needed to Improve Results? New Approach to Analysis of Match Status in Professional Soccer

**DOI:** 10.3390/ijerph16122233

**Published:** 2019-06-25

**Authors:** Marek Konefał, Paweł Chmura, Kacper Rybka, Jan Chmura, Maciej Huzarski, Marcin Andrzejewski

**Affiliations:** 1 Department of Biological and Motor Sport Bases, University School of Physical Education, 51-612 Wrocław, Poland; marek.konefal@awf.wroc.pl (M.K.); kacperliv@gmail.com (K.R.); jan.chmura@awf.wroc.pl (J.C.); 2 Department of Team Games, University School of Physical Education, 51-612 Wrocław, Poland; 3 Department of Team Games, University of Rzeszów, 35-010 Rzeszów, Poland; huzja@op.pl; 4 Department of Recreation, University School of Physical Education, 60-101 Poznań, Poland; andrzejewski@awf.poznan.pl

**Keywords:** team sports, notational analysis, shots, passes, match results

## Abstract

The aim of the research detailed here has been to assess the frequency with which football players engage in technical activity of various different types, in relation to seven phases of a game associated with changes in match status. To this end, 2016–2017 domestic-season matches in Germany’s Bundesliga were analyzed, the relevant data being retrieved using an Opta Sportsdata Company system. Technical activity taken into consideration included shots, passes, ball possession, dribbles, and tackles. It was found that there was a large impact of frequency of shots on target (H = 466.999(6); *p* = 0.001) in relation to the different match-status phases. Furthermore, moderate effect sizes were then obtained for frequency of shots (H = 187.073(6); *p* = 0.001), frequency of passes (H = 133.547(6); *p* = 0.001), and percentage of ball possession (H = 123.401(6); *p* = 0.001). The implication would be that a team trying to change the match score of a game experienced at a given moment in a more favorable direction will need to raise the frequency and accuracy of passes, the percentage of ball possession, and the percentage of tackles ending in success. The maintenance of a winning match status requires a high frequency of occurrence of shots and shots on target as well as greater frequency and effectiveness of dribbling. The main finding from our work is that consideration of the consequences of a game presented in relation to seven potential phases to match status can point to a novel approach to analysis.

## 1. Introduction

Notational analysis is an integral part of modern soccer [1]. Appropriately processed data are helpful, and of practical value, to coaches as they manage, because they make it possible for objective, clear and concise information to be transmitted to players [2,3,4]. The high dynamic and multidirectional character of today’s soccer matches requires detailed match analysis with modern, technologically-advanced motion analysis systems [5,6,7,8,9]. Furthermore, the high level of validity and reliability of these systems denotes many possible applications in scientific research connected to the technical and physical activity [10,11,12], or the tactical play, [13] of professional soccer players [14].

Success in soccer is conditioned by tactics based on appropriate levels of physical and technical activity [15]. However, in the match context, technical indicators prove to be more accurate predictors of success than those of a physical nature [8,16,17,18,19]. The predominant technical activities subject to analysis in scientific publications are shots, shots on target, numbers of passes, and pass accuracy, but the interest in these reflects their being linked most readily with match outcome. [20,21,22]. Lago-Penas et al. [23] observed that teams scoring first goals won approximately 70–75% of all their matches. In turn, Konefał et al. [12] found that, when the number of passes players make during a match is greater by 1, it is associated with a 3.3% greater chance of victory for this team. The execution of a large number of accurate passes affects ball possession, which is most often determined by the current score-line [24]. Furthermore, Link and de Lorenzo [25], looking at other technical activities of importance in soccer, found that the key technical activity engaged in by German football players takes the form of duels and tackles.

However, the analysis most useful when it comes to implementation in sports practice would seem to involve not the number of technical activities engaged in by players, but their frequencies of occurrence and duration. Reference to this could then determine the application of appropriate frequencies of occurrence of technical activities in training games also [26]. Reference to the frequency of occurrence of different kinds of technical activity also allows for analysis of results in relation to contextual variables whose persistence through the time a match is played is not equal, e.g., time spent winning, losing, or in the circumstances of even scores [26].

It has been reported previously that match status [17], but also match outcome [23,27], and position in the table at the end of the league season [28], can be deemed to be related most significantly to team performance in the course of a soccer match [29,30]. The development of soccer players’ efficiency therefore requires continuous analysis of their technical activities affecting the score [31]. However, it would seem that the most interesting and up-to-date is a conceptualization of players’ technical activity in the context of match status, as an early goal or the first goal in a match can clearly influence, not only the final match score, but also the way a team plays the entire game; and that team’s match objectives [32,33]. Thus far, match status has resolved into the basic contextual variables of winning, drawing, or losing, and it was shown in this context that the phase of the game in which players enjoy the status of being the winning team sees them take more shots, make fewer passes, and spend less time in possession of the ball than the team that is behind in terms of score [26,34]. To better understand the routes leading to a win, it is necessary to assess what happens in the course of a match, and the consequences of any given kind of activity engaged in by players [30].

However, to the best of our knowledge, the scientific analysis of technical activity carried out to date has confined itself, when dealing with match status, to the aforementioned conceptualization based on the current state of play (with a team deemed to be either winning, drawing, or losing at any given point). This means that no attention was actually paid to the consequences of play in line with a given status. It is possible to recognize that the consequences of a defined means of playing, while the status of winning is already in place for a team, may involve the maintenance of that status, or else loss of that status, with conceding a goal ensuring a return to the status of “drawing”. Indeed, there is always the possibility of a changeover to the status of winning, drawing, or losing.

In this way, the consequence of actions taken by players in terms of their activity may be for the status of losing to be maintained, or else for a return to the status of “drawing” being achieved. This brings us to the statement that match status allows us to deal, not with three but with seven different results arising out of seven phases of a match in which play leads to either a change of status or to the ongoing maintenance of the existing status. Beginning with the most favorable circumstances from the point of view of the rules of league matches, we have the possibility of points totals rising as the status of drawing gives rise to that of winning (given that a draw earns 1 point, while a win is worth 3). Then we have the phase in which “trailing” status is lost and drawing starts (and there is thus a move from 0 points acquired to 1 for the draw). Then there is the issue of maintaining the number of points, with winning continuing (winning to winning) or else drawing or losing (i.e., drawing to drawing, losing to losing). Ultimately, we come to the changes of status involving the loss of points, as transitions of the drawing to losing and winning to drawing kinds are made.

Soccer teams change their game style with regard to the score line [30]. It was thus interesting to consider whether such a style change in terms of the frequency with which various kinds of technical activity are engaged in by players would be reflected in change as regards the conceptualization of match status as described above. The aim of the research detailed here has thus been to assess the frequency with which players engage in different types of technical activity, in relation to seven aforementioned phases of the game, that resulted in status changing or being maintained in matches played out it in the 2016–2017 domestic season in Germany’s Bundesliga.

## 2. Materials and Methods

### 2.1. Sample

Three hundred and six matches of the 2016–2017 domestic season in Germany’s Bundesliga were analyzed. In order for the objectives of the work to be achieved, match status needed to be divided into seven phases leading to either a change in―or the maintenance of―the result of the match at the given moment. Phase 1 (represented by 380 observations) is a phase in which a drawn game experiences a change of result in the direction of a win (D→W); phase 2 (155 observations) is in turn one in which a team trailing in a match achieves a change of result in the direction of a draw (L→D); phase 3 (575 observations) is one in which a winning result goes on being maintained (W→W); phase 4 (149 observations) involves the maintenance of a draw situation (D→D) and phase 5 (571 observations) sees a team that is behind in terms of the score continuing in that situation (L→L). Phase 6 (characterized by 379 observations) is in turn a phase in which a match standing at a draw heads in the direction of a loss for one team (D→L), while phase 7 (151 observations) sees a change in the current result from one in which a given team is ahead back to a draw situation (W→D).

The study was conducted in compliance with the Declaration of Helsinki and was approved by the local Ethics Committee (No. 339/15). The Study Protocol was also approved by the Board of Ethics of the University School of Physical Education in Wrocław.

### 2.2. Procedures

Data were retrieved from the WhoScored.com website (www.whoscored.com), which uses data resources provided by the Opta Sportsdata Company [22]. The software used by Opta Sportsdata is the Opta Client System which can generate live match statistics. Every possible type of ball touch and on-the-ball action in a match is covered by a rigid set of definitions recorded in the system [35]. Every action requires a player to be assigned to it, along with a time (mm: ss of match time) showing when the event actually happened. All players’ ball touches and on-the-ball actions and their time of occurrence can be recorded and used to generate output from the system. Team actions (other than “ball possession”) constitute the sum of activity of all individual players. Ball possession is calculated as the amount of time a team possesses the ball during a game [14,22]. The inter-operator reliability of the company’s tracking system (Opta Client System) used to collect match statistics was identified as on an acceptable level [14]. Furthermore, Liu et al. [14] showed that team match events coded by independent operators using this system reached a very good agreement (weighted kappa values of 0.92 and 0.94), with the average difference of event time is 0.06 ± 0.04 s.

Table 1 details the technical activity engaged in by players that are under study here, as well as their operational definitions [14,35,36,37].

Given that the amounts of match time assigned to the different phases analyzed differed (D→W―26′54″ ± 01′08″; L→D―23′10″ ± 01′33″; W→W―20′02″ ± 00′48″; D→D―43′58″ ± 02′47″; L→L―19′59″ ± 00′48″; D→L―26′47″ ± 01′08″ and W→D―22′55″ ± 01′34″), the types of technical activity engaged in by the players studied were characterized as the frequency of occurrence of performed activities in minutes and seconds (playing time/number of technical activities engaged in) [30]. Where a given technical activity is not noted in a studied part of a match, the arithmetic mean frequency for that activity is taken from other matches [38]. Pass accuracy, ball possession, dribbling success, and tackling success were all expressed as percentages.

### 2.3. Statistical Analysis

The Shapiro–Wilk test and Levene test were used. Arithmetic means, median, standard deviations and standard errors were calculated (the description of the results here presents arithmetic means ± standard errors to allow for comparison with other studies). Differences between categories were calculated using the Kruskal–Wallis H test. Where a significant effect size was found, a post-hoc Conover–Iman test was performed [39,40]. The level of statistical significance was set at *p* < 0.001. Moreover, the partial eta squared (η2H) value was calculated, and the effect sizes determined: <0.05―small effect size; ≥0.05 and <0.14―medium effect size, ≥0.14―large effect size [38]. All statistical analyses were made using the STATISTICA ver. 13.1 software package (from StatSoft. Inc., Tulsa, OK, USA).

## 3. Results

**Figure 1:**
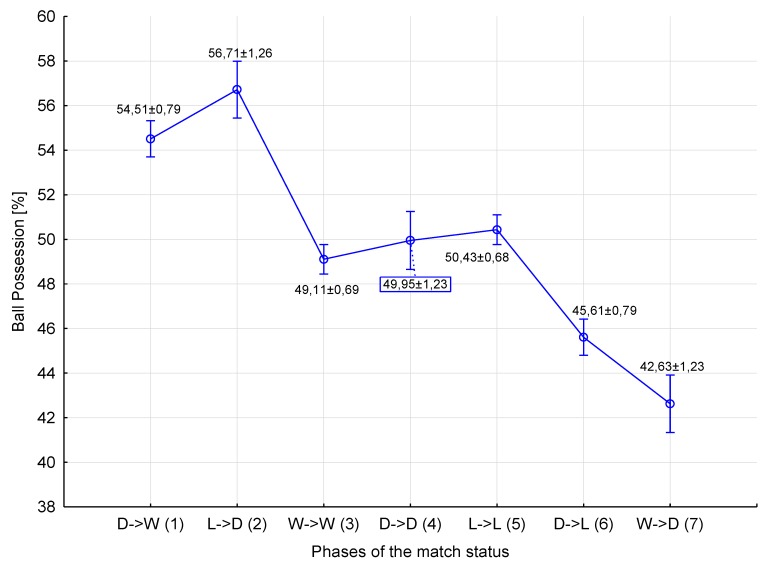
Differences in ball possession achieved by soccer players with regard to match-status phases (mean ± SE). Statistically significant differences: 1,2,3,4,5 > 7; 1,2,5 > 6; 1 > 3,6,7; 2 > 3,4,5,6,7.

The statistical analysis of the frequency of occurrence of technical activities in relation to match-status phases (D→W, L→D, W→W, D→D, L→L, D→L, W→D) revealed effects in relation to the frequency of shots on target (H = 466.999(6); *p* = 0.001)―large effect size (η2H = 0.196), the frequency of shots (H = 187.073(6); *p* = 0.001)―medium effect size (η2H = 0.077), the frequency of passes (H = 133.547(6); *p* = 0.001)―medium effect size (η2H = 0.054), and percentage ball possession (H = 123.401(6); *p* = 0.001)―medium effect size (η2H = 0.050). Furthermore, effects were noted in percentage pass accuracy (H = 63.598(6); *p* = 0.001)―small effect size (η2H = 0.024), the frequency of dribbles (H = 34.985(6); *p* = 0.001)―small effect size (η2H = 0.012), percentage dribbling success (H = 27.599(6); *p* = 0.001)―small effect size (η2H = 0.009), and percentage tackling success (H = 34.116(6); *p* = 0.001)―small effect size (η2H = 0.012) (Table 2, Figure 1).

No significant effect was found for the frequency of tackles (H = 15.773(6); *p* = 0.150)―small effect size (η2H = 0.004) in relation to match-status phases (Table 2).

## 4. Discussion

This study’s provision of precise information on the frequency with which different technical activities are engaged in by players in the context of match status seeks to improve the efficiency of team play as well as to assess the effectiveness of the tactical solutions coaches offer [41]. A novel way of presenting analysis of the technical activity of different types engaged in by players is offered, given the context relating to the different phases of match status. The aim of our research was to assess the frequency of occurrence of player engagement in technical activity of different types, in relation to the seven phases in a game, that either lead to a change in match status or maintain the existing status, as was revealed in the 2016–2017 season in Germany’s Bundesliga.

As our results make clear, players play the longest (43′58″ ± 02′47″) in the D→D phase, which emerges as one in which fewer shots, shots on target, and passes are engaged in, while values for the remaining kinds of technical activity are neither especially high nor especially low. Evidently, this kind of activity suffices in a phase where the status of draw is being maintained (D→D)―with this of course being the initial situation in every match played. Activity engaged in by players at varying levels of intensity during a match can obviously give rise to two possible changes of status―in the D→W or D→L directions. This is in fact a key aspect, as the gaining or conceding of the first goal in a match has a clear influence on the way a team goes on playing a game, and on that team’s objectives, as well as often on the final result [32,33]. Our work makes it clear that shots and shots on target occur far more frequently in the D→W phase characterized by goal scoring than in the D→L phase in which a goal is conceded. This finding is in line with those of an earlier study pointing to differences between professional teams going on to win or lose being mostly visible in terms of the number of shots on target taken, and their efficiency [20]. This is confirmed in analyses of the 2002, 2006, 2010 and 2014 FIFA World Cup competitions [21,22,42], as well as in National League tournaments [43]. Equally, in the group phase of Euro 2016, match status did not have a noticeable impact on the frequencies of occurrence of shots and shots on target, even if―as in our work―the highest frequencies of occurrence of shots (for 08′12″ ± 00′40″) and shots on target (for 23′38″ ± 02′37″) are present when a team is ahead, while the smallest number characterizes the circumstances of a match in its drawn phase―with figures of 10′58″ ± 01′03″ and 28′59″ ± 02′20″ respectively [26]. It may be noted that in the 2016–2017 Bundesliga season analyzed, players were more frequently effective in their shots at the goal than were their counterparts participating in Euro 2016. However, our work’s most major finding is the fact that shots and shots on target occurred with highest frequency while a favorable result for a team is maintained in the context of the W→W phase. Shots on target account for 46.75% more time during this phase than during the D→D phase, for 45.28% more time than with D→L, and even for 41.51% more time than with D→W. This suggests that it is not only the achievement of an advantageous result that is associated with a large number of effective shots at the other team’s goal, but also above all the maintenance of that winning status.

A game involving regular shots may prove risky, though, as our results also make clear. Shots take up a rather large amount of time proportionally in the W→D phase, while only accounting for 20′02″ ± 00′48″ during the W→W phase. It may be that the search for greater efficiency should focus its efforts on the types and proportions of technical activities engaged in during match status phases associated with a move to a more favorable result (i.e., L→D and D→W).

Many studies concerning soccer have focused, not only on the number of shots, but also on the way in which goals are scored [2,44]. The fact that the L→D and D→W phases compare with the others in being associated with significantly more frequent passes and higher pass accuracy as well as greater ball possession suggests that these are important parameters in the Bundesliga context, when it comes to the points advantage over the opponent being extended; thus, representing a correct route to the achievement of a high value for shots. This was confirmed by Bradley et al. [45] and Göral [46], with both studies showing that both numbers of passes and passing effectiveness correlate positively with the result of a match ultimately achieved. Players in all positions make the greatest numbers of passes, and achieve the highest pass accuracy, in matches that go on to be won. The importance of a high incidence of passing in today’s soccer gains confirmation in the finding that, in the World Cup Finals over the period 1966–2010 inclusive, every single variable considered changed significantly over time, while the most major change related to the passing rate [6]. Furthermore, Bush et al. [47] showed that the overall number of passes increased by 40% across seven consecutive seasons of the English Premier League. However, effectiveness in terms of technical activity is more correlated with results than with mere incidences of given kinds of activity [22]. Our research indicates that, in the L→D and D→W phases respectively, teams achieved 76.06 ± 0.58 and 77.45 ± 0.84 percentage pass accuracy, with these values being significantly higher than in phases D→L (70.52 ± 0.74) or W→D (71.03 ± 0.96), both being characterized by changes in results to a situation less favorable for the given team. Such data are similar to those in Dellal et al. [48], in which 70% pass accuracy is cited as the minimum requirement for elite soccer these days―given as a threshold value below which teams most often go on to lose. Barnes et al. [49] showed a trend towards increased effectiveness of passing, stating that the percentage of the occurrence of players with a pass success rate <70% decreased from 26% in 2006–2007 to 9% in 2012–2013.

The execution of a large number of accurate passes affects ball possession, which is most often determined by the current score-line [24]. Lago-Penas and Dellal [34] and Konefał et al. [26] indicated that ball possession was at a higher level when a team was losing than when it was winning or drawing. However, our research shows that, if we take into account, not only the status under which a team plays, but also the consequence of play under that given status, then the greatest percentage figure for possession of the ball is to be noted in the D→W phase (D in the classical conceptualization of match status), as well as with L→D (L in the classical conceptualization). A significantly lower value is obtained with D→L (D in the classical conceptualization of match status) and W→D (classically W). It is worth stressing that, in the classic studies of match status [26,34,50], the D→W phase would have the status of draw, while L→D would represent losing. However, consideration of the consequences of play under a given status presented in relation to seven match-status phases points to a novel approach to analysis.

Analyzing the 1998 World Championships, Grant et al. [51] stated that teams reaching the semi-finals engaged in more dribbling in the offensive play than teams that went out at the end of the group phase. Our work confirms that dribbling remains a key activity among footballers, especially when it comes to maintaining a favorable score―i.e., a lead in a match (the W→W match status). Moreover, our analyses point to the key aspect that it is the effectiveness of dribbling―7.35% greater in the W→W phase (in which a favorable result is maintained) than in the case of D→L (a move in a direction of a less-favorable result ending in a match lost). Also important is the effectiveness of tackling―which is significantly higher in the D→W phase (i.e., a change in the direction of a win from a drawn situation). The percentage of dribbling success would seem to be a key activity for players in defence, and this idea is confirmed by the steady, evolutionary expansion of such activity in successive seasons of Britain’s Premier League, as well as Germany’s Bundesliga [25,49].

Important added value of the present study reflects the proposal of seven different phases of match status. A limitation of the present study is that it is based on one specific domestic league, i.e., the Bundesliga. Further research should involve interdisciplinary monitoring of match status, using highly-selected study data from a top-level sport tournament (e.g., the World Cup, European national football leagues, or the European Champions League), and a greater number of performed technical activities [52].

## 5. Conclusions

Notational analysis as regards to the frequency of occurrence of different kinds of technical activity among teams playing in the 2016–2017 domestic season in Germany’s Bundesliga revealed that, the behavior indicated to help a team change the match status at a given time to a more favorable one (i.e., D→W or L→D) is an increase in the frequency and accuracy of passes, in the percentage of time for which the ball is possessed and in the percentage of tackles ending in success. In turn, the maintenance of the match status involving winning (i.e., W→W) requires a high frequency of shots and shots on target, as well as more frequent―and more effective―dribbling. In turn, a change in match status to a less favorable one (i.e., D→L) is associated with few shots taken and shots on target, as well as a low frequency of occurrence of passes and low pass accuracy, and low percentage figures for possession of the ball and success with dribbling.

## Figures and Tables

**Table 1:** Types of technical activity engaged in by players, and their operational definitions.

Groups	Parameters: Operational Definitions
Shots	Shot: An attempt to score a goal made with any (legal) part of the body, either on or off target. Shot on target: An attempt to score a goal which requires an intervention to stop it going in, or results in a goal/shot which would have gone in had it not been diverted.
Passes	Pass: A ball played intentionally from one player to another. pass accuracy: Successful passes as a proportion of total passes. Ball possession: The amount of time over which a team controls the ball during a game, from the moment the ball is taken on from the opposing team without any clear interruption, as a proportion of total time during which the ball is in play.
Dribbles	Dribble―an effort by a player in possession of the ball to maintain this status as he passes by a player of the opposing team. Successful dribbling denotes retention of the ball. Dribbling success―the number of successful dribbles (as set against all attempts by players to circumvent players of the opposing team).
Tackles	Tackle―an activity entailing the successful obtaining of a ball previously in the possession of a player from the opposing team. Tackling success―the number of successful interceptions of the ball of the above kind as set against all attempts to gain possession of the ball from members of the opposing team.

**Table 2:** Differences in the frequency of occurrence of types of technical activity engaged in by soccer players with regard to match-status phases (mean ± SE).

Technical Activity Engaged In	Match-Status Phases							H (Sig.)	SSD (*p* ≤ 0.001)
	D→W (1)	L→D (2)	W→W (3)	D→D (4)	L→L (5)	D→L (6)	W→D (7)		
shot [1/time]	08′21″ ± 00′15″	08′11″ ± 00′24″	06′42″ ± 00′13″	08′58″ ± 00′34″	08′53″ ± 00′15″	10′39″ ± 00′22″	07′22″ ± 00′23″	187.073 (0.001)	6>1,2,3,4,5,7; 3<1,2,4,5,6
shot on target [1/time]	21′48″ ± 00′40″	19′47″ ± 00′46″	12′45″ ± 00′29″	23′27″ ± 01′25″	18′01″ ± 00′22″	22′45″ ± 00′36″	14′11″ ± 00′55″	466.999 (0.001)	7<1,2,4,5,6; 3<1,2,4,5,6; 3,7<5<1,4,6
pass [1/time]	00′12″ ± 00′01″	00′12″ ± 00′01″	00′17″ ± 00′01″	00′17″ ± 00′01″	00′16″ ± 00′01″	00′16″ ± 00′01″	00′18″ ± 00′01″	133.547 (0.001)	1<3,4,5,6,7; 2<3,4,5,6,7; 6<7
pass accuracy [%]	76.06 ± 0.58	77.45 ± 0.84	73.91 ± 0.66	72.13 ± 1.00	74.45 ± 0.48	70.52 ± 0.74	71.03 ± 0.96	63.598 (0.001)	7<1,2; 6<1,2,3
dribbles [1/time]	06′11″ ± 00′13″	06′42″ ± 00′21″	05′47″ ± 00′10″	05′58″ ± 00′15″	06′28″ ± 00′11″	06′41″ ± 00′15″	05′59″ ± 00′17″	34.985 (0.001)	3<2,5,6
dribbling success [%]	45.18 ± 1.36	46.61 ± 2.10	48.22 ± 1.15	44.42 ± 1.83	44.70 ± 1.19	40.87 ± 1.40	47.87 ± 2.30	27.599 (0.001)	3>6
tackles [1/time]	04′16″ ± 00′09″	04′28″ ± 00′14″	04′16″ ± 00′07″	04′17″ ± 00′14″	05′55″ ± 00′06″	04′16″ ± 00′09″	04′22″ ± 00′13″	15.773 (0.015)	–
tackling success [%]	74.50 ± 1.18	65.05 ± 2.02	70.38 ± 1.02	70.49 ± 1.41	67.58 ± 1.03	69.74 ± 1.27	69.43 ± 1.86	34,116 (0.001)	1>2,5

SSD―statistically significant differences.

## Practical Application

It is recommended that coaching staff keep records of the regularity with which technical activity is engaged in by players during a game in relation to the phases of match status described in this article. Such information allows for the development of team-specific sets of technical activity that should be engaged in more often by players with a view to unfavourable match status being changed or favourable status maintained. Trainers and analysts at training sessions should impose scores during small-sided games, competition matches, tactical drills and game simulations with account taken of the 7 phases of match status and the technical activity detailed by our research. This will in turn ensure the design of training exercises more similar to the actual competition, with teams being able to adapt game style in regard to match status.
